# Mixed fibrolamellar hepatocellular carcinoma

**DOI:** 10.1002/ccr3.4318

**Published:** 2021-06-24

**Authors:** Faten Limaiem, Saadia Bouraoui

**Affiliations:** ^1^ Tunis Faculty of Medicine University of Tunis El Manar Tunis Tunisia

**Keywords:** liver, mixed fibrolamellar hepatocellular carcinoma, pathology, tumor

## Abstract

Pure and mixed fibrolamellar hepatocellular carcinomas display distinct clinical presentations and epigenetic backgrounds leading to different prognoses and as such may be regarded as separate clinical entities.

## Graphical abstract

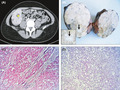

## 1 CLINICAL IMAGE

Mixed fibrolamellar hepatocellular carcinoma is a rare liver tumor defined by the presence of both pure fibrolamellar hepatocellular carcinoma and conventional hepatocellular components.^1^ It represents up to 25% of cases of fibrolamellar hepatocellular carcinoma and has been associated with a worse prognosis.^2^

**FIGURE 1:**
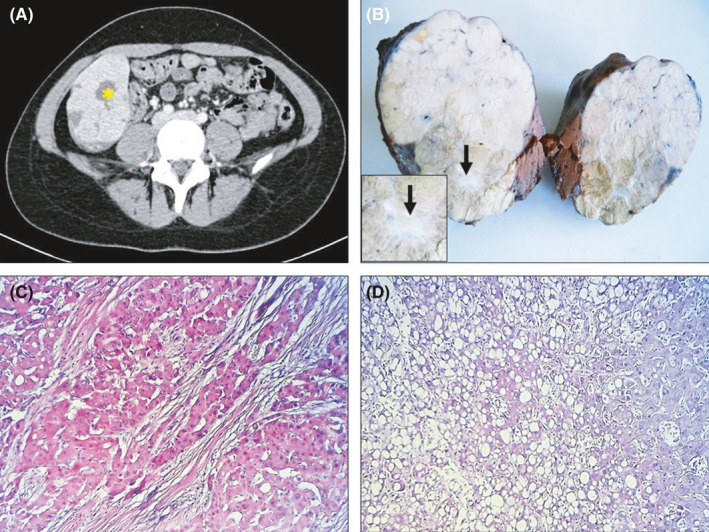
A, Computed tomography scan showing a hypodense hepatic tumor with well‐defined lobulated margins. During the arterial phase of dynamic enhanced CT, the tumor showed prominent and heterogeneous enhancement with a more central portion remaining hypodense (central scar: asterisk). B, Gross photograph of mixed fibrolamellar hepatocellular carcinoma forming a multinodular mass with a central scar (black arrow). Zoomed insert of the fibrous scar. C, Trabecula of neoplastic cells with abundant oncocytic cytoplasm in a background of dense collagen bundles arranged in parallel lamellae (Hematoxylin and eosin, ×200). D, Steatohepatitic hepatocellular carcinoma. The tumor cells in this variant show steatohepatitis with macrovesicular steatosis, lymphocytic inflammation, balloon cells, and pericellular fibrosis (Hematoxylin and eosin, ×200)

A 22‐year‐old previously healthy female patient presented with an abdominal discomfort for the past 2 months. The physical examination revealed a firm nodular mass in the right flank. The serum alpha‐fetoprotein level was equal to 1.48 ng/L (normal <3 ng/L). Nonenhanced CT scan disclosed a hypodense well‐defined liver mass in the right hepatic lobe. During the arterial phase of dynamic enhanced CT, the tumor showed prominent and heterogeneous enhancement with a more central portion remaining hypodense (central scar) (Figure 1A). A liver biopsy was performed and histopathological examination established the diagnosis of hepatocellular carcinoma. The patient underwent liver resection. Grossly the tumor was well‐delineated and lobulated with a central fibrous scar (Figure 1B). Histological examination of the surgical specimen showed a biphasic tumor proliferation of large cells containing large nuclei with abundant and eosinophilic cytoplasm. The tumor cells were arranged in a trabecular pattern and were segregated by deposition of fibrous connective tissue, with lamellae formation (Figure 1C). The second compartment of the tumor was consistent with steatohepatitic hepatocellular carcinoma (Figure 1D). The tumor cells in this compartment showed macrovesicular steatosis, lymphocytic inflammation, balloon cells, and pericellular fibrosis. Postoperative course was uneventful, and the patient has no recurrence at 2‐month follow‐up.

## CONFLICT OF INTEREST

None declared.

## AUTHOR CONTRIBUTIONS

Dr FL and Pr SB: prepared, organized, and wrote the manuscript. Dr FL: performed the gross and microscopic pathologic evaluation of the pathology specimen. She prepared all of the histology figures in the manuscript. She read, edited, and approved the final version of the manuscript. Pr SB: edited the article and revised it critically for important intellectual content.

## ETHICAL APPROVAL

All procedures performed were in accordance with the ethical standards. The examination was made in accordance with the approved principles.

## Data Availability

In accordance with the DFG Guidelines on the Handling of Research Data, we will make all data available upon request.

## References

[1] Malouf GG , Brugières L , Le Deley MC , et al. Pure and mixed fibrolamellar hepatocellular carcinomas differ in natural history and prognosis after complete surgical resection. Cancer. 2012;118(20):4981‐4990. 22415897 10.1002/cncr.27520

[2] Barreira JV , Silva N , Parmanande A , et al. Fibrolamellar carcinoma: a multimodal approach. GE Port J Gastroenterol. 2020;27(6):429‐433. 33251292 10.1159/000507201 PMC7670382

